# Dermatoglyphics in childhood leukaemia: a guide to prognosis and aetiology?

**DOI:** 10.1038/bjc.1978.154

**Published:** 1978-06

**Authors:** M. Till, S. Larrauri, P. G. Smith

## Abstract

The results of analysis of the dermatoglyphics of 152 children with acute lymphoblastic leukaemia (ALL) (and the first-degree relatives of 54 of them) contrast with those of 31 children with acute myeloblastic leukaemia (AML) (and the first-degree relatives of 25 of them). In ALL our findings suggest that neither genetic susceptibility nor an environmental factor, effective during the early antenatal period, is of aetiological importance; but the response to treatment, assessed as length of first remission, was found to be related to the amount of fingertip pattern. This may have clinical application. In AML there is evidence of a genetically determined factor carrying a high risk of the development of the disease, in that a member of each of 5 different families of the 25 studied bore a rare hypothenar pattern, compared with none in 75 control families. No dermatoglyphic features were of prognostic significance in AML.


					
Br. J. Cancer (1978) 37, 1063

DERMATOGLYPHICS IN CHILDHOOD LEUKAEMIA: A GUIDE

TO PROGNOSIS AND AETIOLOGY?

1\1. TILL*, S. LARRAURI*P AND P. G. SAMITHt

Fronim the *Departnient of Haematology, Institute of Child Health and Hospital for Sick Children,

London WVC1N 3JH, and the tDHS8 Cancer Epidemiology and Clinical Trials Unit,

Department of Regius Professor of Ml/edicine, Un iversity of Oxford, OX2 6HE

Receive(d 26 January 1978 Accepte(d 16 Februiary 1978

Summary.-The results of analysis of the dermatoglyphics of 152 children with acute
lymphoblastic leukaemia (ALL) (and the first-degree relatives of 54 of them) contrast
with those of 31 children with acute myeloblastic leukaemia (AML) (and the first-
degree relatives of 25 of them).

In ALL our findings suggest that neither genetic susceptibility nor an environ-
mental factor, effective during the early antenatal period, is of aetiological import-
ance; but the response to treatment, assessed as length of first remission, was found
to be related to the amount of fingertip pattern. This may have clinical application.

In AML there is evidence of a genetically determined factor carrying a high risk of
the development of the disease, in that a member of each of 5 different families of the
25 studied bore a rare hypothenar pattern, compared with none in 75 control fami-
lies. No dermatoglyphic features were of prognostic significance in AML.

THE NUMBER and arrangement of derm-
al ridges on human hands and feet provide
a permanent record of events during the
first 4 months of foetal life, during which
time the early development of the thymus
and immune system also occur.

These features are mainly genetically
determined, but may also be affected by
other factors (such as exposure to terato-
gens or maternal infections) which inter-
fere with foetal development during that
period. The possibility that environmental
influences during early foetal life, in
addition to a possible genetic immuno-
deficiency, might be of aetiological import-
ance in childhood leukaemia, prompted the
study of dermatoglyphiics in patients with
this disease.

The conclusions of the 3 largest pub-
lished series confined to acute lympho-
blastic leukaemia (ALL) in children are
conflicting. Berka et al. (1971) found no
dermatolglyphic features to distinguish a
series of 50 patients from controls;

Purvis-Smith and Menser (1973a) found
evidence of predisposing genetic factors
only, in a series of 135 patients and their
relatives, whereas Wertelecki et al. (1973)
who published material from 76 patients
and their relatives, suggested that in
addition to a genetic factor there also
appeared to be some antenatal environ-
mental influence distinguishing male
patients from their brothers.

The distinction between genetic and
environmental antenatal influence is of
importance aetiologically and requires
clarification.

SUBJECTS AND METHODS

Dermatoglyphic prints were obtained by
the Faurot technique from hands and feet of
152 Caucasian children (87 boys, 65 girls)
under the age of 15 years in whom a diagnosis
of acute lymphoblastic leukaemia (ALL) was
made at the Hospitals for Sick Children
between 18 May 1972 and 31 December 1976.

+ 'resenit a(l(lress: hist ituto Venezuel() (de Tnvestigaciones Ci(entificats, Caracas, Ven.ezuela.
+                                                             I

(19

M. TILL, S. LARRAURI AND P. G. SMITH

Patients previously treated or referred for
advice before starting treatment elsewhere,
were not included. There was no case of
Down's syndrome. In total only 17 patients
(10 boys, 7 girls) presenting during this 4-
years, were not printed, thus the series
includes 90%  of Caucasian patients newly
diagnosed as ALL during this period. Four-
teen of those not printed died within 2 years
of diagnosis, 5 of them without remitting.
Three are still alive: one is living abroad, one
is too young to print satisfactorily and one has
gross congenital deformities of the hands and
feet. The first-degree relatives of the last of
these are, however, included in the series.
Amongst the children included in the series
were 11 (10 boys and one girl) whose blast cells
form E rosettes: 9 of these were classified as
T-cell ALL and 2, who presented with media-
stinal mass and pleural effusion but without
overt leukaemia, as T-cell lymphoma. Blast
cells with T-cell markers were plentiful in the
pleural fluid of these 2 patients, but neither
had subsequent involvement of the blood or
marrow. One other patient had B-cell
leukaemia.

All the patients remitted. The majority
were treated according to one of the Medical
Research Council's trial schedules; all receiv-
ed similar induction protocols followed
by a course of radiotherapy to the central
nervous system soon after remission was
established. Maintenance schedules varied
with the trial, the more intensive regimes
being reserved for those presenting with high
leucocyte counts or T-cell ALL leukaemia,
but all received essentially continuous main-
tenance therapy with multiple drugs at
maximum tolerated doses. Treatment was
stopped, according to schedule, in patients
who were still in first remission, either at 2
years or 3 years after diagnosis.

Prints were also obtained from first-degree
relatives of 54 of the patients who presented
during 1974 and 1975. These were families of
49 consecutive new patients with ALL (5 with
T-cell ALL) where both parents were avail-
able and willing to take part in the investiga-
tion, together with 5 additional families of
patients who presented subsequently with
T-cell ALL. In these 54 families there were 73
(35 male, 38 female) siblings of the patients;
but only 18 male and 5 female patients had
like-sexed siblings. Each family provided a
"control" family, usually friends of theirs,
matched as nearly as possible for age of the

parents and including a child of similar age to
the patient.

The dermatoglyphics of 31 Caucasian
children (19 boys, 12 girls) with acute myelo-
blastic leukaemia (AML) were also studied
together with the parents and siblings (16
male, 12 female) of 25 of them. These consti-
tute two-thirds of such patients who pre-
sented at the Hospitals for Sick Children
between 18 May 1972 and 31 December 1976,
and are cytologically a heterogeneous group:
23 were myeloblastic, 5 myelomonocytic, 2
monocytic and one erythroblastic. Control
families were matched for only 21 of these
and, together with those matching the 54
ALL families, included 145 children (68 male,
77 female).

Interpretation of all areas of the prints
(excluding toes) was carried out using the
nomenclature for loops on palms and soles de-
scribed by Penrose and Loesch (1969, 1970).

Standard measurements were made, which
included total ridge count (TRC), a-b count
and maximal atd angle. Pattern intensity on
the fingers (PIF) was scored as the sum of
triradii on the 10 fingers, and pattern
intensity on the palms (PIP) and soles (PIS)
as the number of loops on both palms or both
soles. Total triradii (TT), being the total
number of triradii on both hands and feet but
excluding toes, was also calculated.

In addition to the dermatoglyphics, palmar
creases were also studied. The scoring of
abnormalities of the palmar creases is very
subjective, owing to the wide range of
aberrant forms. A classical simian line was
scored as such and aberrant forms of it
separately. A Sydney line was only scored if
the proximal palmar crease reached the ulnar
margin of the palm. No aberrant forms were
scored. The prints were read independently by
2 of us and then all were read "blind" a
second time. Only those creases consistently
reported as abnormal were scored.

Results were compared using the x2 or
Student's t test as appropriate. In composing
groups of control boys and girls for com-
parison with patients of like sex, children of
both sexes were used from every control
family where they were available. In order to
obviate any possible bias due to over-
representation of large families, not more than
one boy and girl from any one control family
was included, those selected being those
nearest in age to the patient to whose family
the control family was matched.

1064

DERMATOGLYPHICS IN CHILDHOOD LEUKAEMIA

o       o  t0

0- Qk 0o  0  -  @  _

? a,)   00 co co N  xo

o      w4C

o ~ ~ o ~   0

X o!e o    A_; A Q

-     - t   a

aq 0-

to  aq  ;     -  S o

u  So  <  <  X  > to  v

0C  e ' c   0-C  -

s              *^  e~~~~~

0 ,    I d)4  -O CsC   i

0~~~~~~~

0   C>   0,  C; Co  o   C > 0

E ~~ ~             ri " ?   l 2  *

0

<>   _  ~~~m co   U

-             ri2~~~~~~~-

00  10

-C C    - - - (   0

~~0 0 00  ) C 0 0 1 to

Eo o' i -    -   x

V 2   0 M a   , ~ ;

Zs         ~          C
pq ~ ~ ~ ~   C  b C)

1 0 1~~~~

co&o       C)~~~~4
p~~~~~~~.. ~ ~ ~ ~ ~ A

1065

M. TILL, S. LARRAURI AND P. G. SMITH

0 1 0 -   CO~   - 4

0101)     COO 01

" o) " CD m - t- o

- - - -   N-   00 =

-4 - -4 -4 t-

- CO N O b 4  O  CO

- 0    CO C-  0   0   -

0 t cO
r- t- C

1 00

- - -, .-

co _q - q o  e1

001010a   01  CO
00  0 01 C

".4   -*10  -*  01 9   I  4   -4  10

N-   -4   04   "   a . Q%N -  -   CO

O  Nt   10   0 1   M

- -"  C -   C)  -  a'10 c 0  0   -l- --.

0 0 0 1  CO  - O 1k 10  CO   0   0 O  _

C   0 " a  q =0   q I   a00   0)  C O 0 00C  0   COm

0C   "-4  C  OCO  -   N N-  0-  =  01   I

- - - -4 -      -  - (= -4

N CO -co       t-   4 O

0 1 O    c C oo       O CO d  m  t
P- N   oo (      co =

" " aq aq 00

_0c0        CO _

m     CD _   1001

in VD m     to co r-

01CO     00  0=

CO   0 L 'o ltN

COOQ

N U-:10C

-0   O    4 C O 0

CO   10   - CO

01     10 CO0

e  C - O = es  es q   t- c - 10

_q410   Co  O  w  1

P--4   COO  -4  N Nt-t

_   0 1 0 0 1l   C O O   C

_ _ _   _      _ -  0  0

-C) -0 -

0-4 0 c - oo

0 co    CON qt-

0 -00001  - 01N00

?

0-

- - -   -

aqt  - m    N

1 0 -0- C      -

0   0 1N4  o   0

-   -  COCO  -

CO0-  -~  0    -

10- c     oo   0

0 1 0   C O O (   - 4

- C - -

-10 - 10

0101q

-4 N-
ic__1t

F-410  N-

-01 0
CO" =

r to co

10 0 N

0 N

10      -4"- 1010
0 0   -     c

c co    0-  t-t

*

*

0      - -

c4  010 10 -  C

- aq aq (=  -

0  CO     C

;l    -

CO  t-I   -

w cow 0 O

4    4   0   1

10   *

-4 " 4 1 1 -   CO

N  C O 1 0-   -   0  CO

b  01CO e o   c

C O  - 0 1 1   0 1 C

01  C O -t  t O   s-

CO   0 1 01-   -

-  - -  -  CO  -  CO

b4  0 1 C   _o

O
1  0

- 0

W C)M

X      <  ~  .4  2

* ; W o B E g = S++

0Z m   t

-

0

0

0

0
0

1-

W

0

0

w
C4-'

0
._
0 0

0
W
C'
0

CO
W
.0

0_
M0

0^

M
W0
0
0

1._

0
0

bO

0._
.0*_

1066

-4

;-4

0

o

4    04.

0 M

S~   O2

O4z

ez V

0 0 10
0

<     ? o

4 O

14.4

oo

Cl)

S

1:

Zs

Vp
V4

Iq

DERMATOOLYPHICS IN CHILDHOOD LEUKAEMIA

17--

" 't

-4- -;.. mt
- a L.

't
(:) r.,

d)

Cer        10;
cV 0+ t-

O0?

t 0e

4 -

_

.ts Y & I O

Vt'

~a - Z; ,.

Ct     _
V

o        =t

0>

-z

EH

.C

S

z5

X  I-_
01     1<

-  1.^  0  d!1
10  10  10
_       10

0

_       CO

N ~

-       CO

0 C  COe

_-0   0 4

G ONt

01

r."  -

01   "

- -

-

01   -

0    01

I- -
C]   -

I-1

01t

e,.  -
OC   -

LI:  t-

aA

-   e.1
0   "
-   1

01-

-   il:~

C   N

0-
C t

-   It

1'-

e   t_

01_

-   C--

C   =

if:

1067

;710

-4

N.:

10

10
S

CO

10

00

-4

t-

- l

_    C_

tC
-4

C..

00   01

-    C"

00  OCO

_     _
-     _1

0    01
- C0

N _4

-    CO
_   e4
_-    _

-   O.

CC O

_O _

N- G

0 .1

CO CO

CO

_ 01
- _O

C1 C

_ CO

- 01

00 C:O

C 01
oO CO

1  01

00 CO

CO

00 e:

10 N

10 CO

N 10

- c
10   -

CO

_ 00
I- 01

10 c
_O -
_    -_

00    c

100
N    0
lO

=    -o

01   00
N    -
*74  o0
t-    -q

10 N
41:  t-

t-

_0 _

N0 01
t-  (C,

C:   O-C

CD 0

_ _-

t-   CO

N _1

O    00

CD   -

00   01,

10   CD
CD   -

t -  c.'

01 0

00   CO

10: -
- -
CDC

CO -

N    -
C- -
00   01

00

CO
CO

CO
CO

M

01

00

t-
I1
N
CO

-1
CO
.

t-

v

1-5

01

1-
-4

0

CC
-
-4

-
0-

U:4

CX

C,

,~ 3:-      t-

C,

_~     *     .Y
-_   if:    1 _

" C70       '
.1 10 N

I,    1     N

_' t' -
- VOC

?O X '

000

cO

O1 '^ 00

.N .

co

0

_ . .0
l _CX

C-

OC  '- *  !

~~~~-q

I.  .Y CZ O

G  - 0

1

4

I
I

M. TILL, S. LARRAURI AND P. G. SMITH

RESULTS

ALL patients

Comparison of the findings in the ALL
patients with those of both control
children and control parents of like-sex are
shown in Tables I, II and III. In Table I
the mean ridge count (TRC) for female
patients (134) is significantly (P<O005)
higher than that for the girl controls (116)
but it scarcely differs from that for control
mothers (131). In Table II, the proportion
of male patients with third interdigital
loops (74%) is significantly higher than
that in control fathers (57 %) but is close
to that in control boys (76%). Amongst all
the features listed, the values for ALL
patients differ significantly from those of
controls in only these 2 instances. The
inconsistency of these two differences when
control parents and children are used for
comparison suggests that they probably
arose by chance. Sydney lines (Table II)
occur more frequently in male patients
than control boys but not significantly so.
No similar difference is seen between fe-
male patients and controls. The normal
sex differences (Cummins and Midlo, 1942)
which are in the main well demonstrated
by the controls, are not consistently appar-
ent among the patients. The expected
male excess of digital whorls and radial
loops is present, but the higher mean TRC
in males and the excess of arches in fe-
males are absent. The mean values for
TRC, PIF and digital whorls for the 10
patients with T-cell ALL are 130-5, 12-5
and 33 respectively. These do not differ
significantly from the values for the other
142 ALL patients.

These results are similar to those of the
small series of patients reported by Berka
et al. (1971) who found no significant
differences between patients and controls,
and do not support the finding of in-
creased prevalence of digital whorls and
raised PIF in the 2 other series of patients
(Purvis-Smith  and    Menser,   1 973a;
Wertelecki et al., 1973). It is possible that
these apparent differences between series
might be resolved if digital whorls and PIF

were found to be associated with some
other characteristic which was differently
represented amongst the patients in the
various series.

Dertnatolyphiesandprognosis.-The mean

1. 0                 DIGITAL WHORLS
0.18 -.OIo '----------------  On-51)

0.6   I 1or 2n-42  L
0.6

0.4 [

0.2

z

- 1.0

0.8
0  O0. 8

0.6
0.
-0.

1L 0. 6

0

0.

-  0. 2

1. 0

0.8
0.6

0.4 P

a.2

L.           PATTERN INTENSITY FINGERS

11 ,            0-11An-75)

.I  --- ---

I_ ,

1  I---In- I

15t   in.33)  ,1  '

I  I.------... .-- -_-

15- _ --1- I  _ _ _

-<, ......... TOTAL RIDGE COUNT

<l1OOn-36)
I

,    L O TA - .

IL____- (n-571

150+{n-591 L  __ _ _ _

111

i?-

O       1       2        3       4       5

TIME SINCE DIAGNOSIS Iyearsl

FIG.-Length of first remission in patients

with ALL according to dermatoglyphic
characteristics. The curves are biased
towards overestimating the time to first
relapse in each of the groups, as they do not
include data on 12 patients who presented
for treatment in the period 18 May 1972-
31 December 1976 and who achieved clinical
remission. Nine of these patients died
before dermatoglyphic studies could be
performed (these patients died at 14, 16, 19,
19, 26, 32, 40, 53 and 64 weeks after
diagnosis). One patient emigrated 93 weeks
after diagnosis. One patient is too young to
obtain satisfactory dermatoglyphics and
one is congenitally malformed and has only
rudimentary fingers. Five additional
patients did not achieve clinical remission
and are also excluded.

I                                                 I         -                                       I                                                  I                                                 I

1~~~~~~- -- 1  1

I~~ I I

1068

--------            I.-

59)  L

3+ fn-     L -        L---------

L - - - - - - - - - -

DERMATOGLYPHICS IN CHILDHOOD LEUKAEMIA

TABLE IV.-Digital Pattern Indices in ALL Patients According to Age at Diagnosis

Age at diagnosis

< 3 yrs
3-5 yrs
> 6 yrs

WBC at
diagnosis

(109/1)
<5 0

5 0-20
> 20

27
27
33

ALL PatientS (bOth SeXeS)
Number

o                 T

d         S?      Total      TRC

46
53
53

19
26
20

133 0
137 9
126 0

TABLE V.-Digital Pattern Indices in ALL Patients According

to Leucocyte Count at Diagnosis

ALL patients (both sexes)

I                '

Number

d          y        Total
31         26        57
28         30        58
28          9        37

TRC
122 0
137 9
140-4

TABLE VI.-CoMparison of Dermatoglyphic Features in ALL Patients According to

Response to Treatment

No. of patientst
Mean TRC

Mean % fingers with digital whorls

ulnar loops
Mean PIF

% hands with interdigital loop III
(% of persons with loop on 0,1 or 2

hands)

Death or relapse within 2 years  Survival 2 years from diagnosis

of diagnosis                without relapse
35 (26, 9)1                   72 (34, 38)

142 (143, 140)                126 (115*, 136)

36-3(36-5,35.6)               21-5*(19-7*,23.2)
54.9(54-2,56.7)               66.9*(63-8,69.7)
13-1(13-1,13-0)               11-7*(11-2,12-1)

60-0 (71 -2, 27 8)            39-6** (41-2**, 38 2)

(26, 28, 46)

(43, 35, 22)*

*P<0.05
**P<0.01

t omitting 45 patients who were in their first remission but had survived less than 2 years at the end of the
study.

$ (males, females)

values for digital whorls, PIF and TRC for
patients in this series, listed according to
the patient's age and leucocyte count at
diagnosis in Tables IV and V respectively,
show a tendency towards increased pat-
tern in younger patients and in those
patients with high leucocyte counts.
Although none of these differences reach
statistical significance the trends are
present in both sexes. Younger patients
and those with high initial leucocyte
counts are known to have a poorer prog-
nosis, and therefore the dermatoglyphic
features of patients who relapsed or died
within 2 years of diagnosis (while still
receiving chemotherapy) were compared
with those who survived at least 2 years
after diagnosis without relapse (Table VI).
The 35 patients in the relapsed group

include 9 with T-cell leukaemia, 1 with B-
cell leukaemia, and 12 others with leuco-
cyte counts greater than 20 X 109/1 at
diagnosis. There is a significant difference
between the 2 groups for digital whorls,
ulnar loops, PIF and interdigitial loop III
which remains even after omission of the
data from the 9 patients with T-cell
leukaemia. The difference in TRC is
significant if boys are considered alone,
and in general the differences are more
marked in boys. The results in girls,
lhowever, show the same trends, except in
the incidence of interdigital loop III. These
differences, in which increased finger-tip
pattern expressed either as digital whorls,
PIF or TRC, appears to be associated with
poor prognosis, are more effectively de-
monstrated by including the whole group

Dig. Wh. %

-33 0
23 0
22 8

PIF
12*8
12 0
11 6

Dig. Wh. (%)

22 8
27 5
28 9

PIF
11 8
12 4
12*3

1 069

.. l- -

M. TILL, S. LARRAURI AND P. G. SMITH

of 152 patients in life tables for the length
of first remission (Fig.). The trend of
decreasing length of 1st remission with
increasing pattern is statistically signifi-
cant for digital whorls (P = 0.02) and PIF
(P = 0 02) but not for TRC (P = 0-13).
No other dermatoglyphic features were
found to have any significant association
with prognosis.
ALL families

Parents.-Comparison of the dermato-
glyphic features of the parents of children
with ALL with those of the control parents
is shown in Tables I, II and III, the only
significant difference being the higher
prevalence of digital arches in ALL fathers
compared to control fathers (Table I). Six
of the 54 ALL fathers have 4 or more
arches, compared with none of the 75
control fathers. The prevalence of any
abnormal crease in ALL fathers is lower
than that in control fathers and the pre-
valence of the hypothenar loop H is higher
in ALL mothers than in control mothers,

but none of these differences is statistically
significant (Table II).

No significant difference was found
between the dermatoglyphics of the
parents of patients with T-cell ALL and
those of the parents of other ALL patients.
Comparison of the parents of 22 ALL
patients who relapsed or died within 2
years of diagnosis with the parents of 30
patients who had survived 2 years since
diagnosis without relapse, showed that the
mean values for both mothers and fathers
for TRC, digital whorls and PIF are very
similar in the 2 groups. However, the
number of parents included in the study is
considerably smaller than the number of
patients and, indeed, if the analyses shown
in Table VI are restricted to those children
whose parents were also studied, the
differences are no longer apparent.

Children.-In the families of both the
patients and the controls, the proportion
of children with abnormal creases is higher
where a parent has an abnormal crease,
and the difference is significant if cases

TABLE VII.-Prevalence of Abnormal Palmar Creases in Parents and their Qffspring in

Patients' and Control Familiest

ALL families

Neither parent has abnormal

crease

One or both parents have

abnormal crease
CONTROL families

Neither parent has abnormal

crease

One or both parents have

abnormal crease

Total numbers of

Families Patients Sibs Children

Numbers with anomalous

crease

,       -        -   nA

Total

Patients Sibs children

o/
/O

32      32      42     74          7      6      13       17-5
21      21      29     50          8      8      16       32

38

77

34

AML families

Neither parent has abnormal  11

crease

One or both parents have

abnormal crease            14
Combined data (cases and controls)

Neither parent has abnormal

crease                     81
One or both parents have

abnormal crease            69

13       1658
21       30-9

68

11     13     24         4      0      4       16-6

14     14     28          4      3       7       25

30       17-1**
44       30.1**

t One ALL family omitted where patient has congenital deformity of hands.

146

1070

175

I

DERMATOGLYPHICS IN CHILDHOOD LEUKAEMIA

and controls are considered together
(Table VII). This finding is unrelated to
the sex of the parent or offspring, or to the
type of crease. There is no significant
difference between  the prevalence  of
abnormal creases in patients and that in
their siblings of either sex.

Comparison of values for TRC, digital
whorls and PIF between the patients (18
male and 5 female) and their like-sex sibs,
the 36 male patients and their fathers and
the 17 female patients and their mothers
are shown in Table VIII. None of the
TABLE VIII.-Comparison of Dermato-

glyphic Features in ALL Patients and
their First-degree Relativest

Male patients
Their brothers

Female patients
Their sisters

Male patients
Their fathers

Female patients
Their mothers

No.
18

18t
5

5t
36
36
17
17

Mean
TRC
148
166
142
114
142
132
134
142

Mean

Dig.Wh.

44
50
28
23
36
33
24
26

Mean
PIF
13-8
15-1
12-8
11-4
13 -0
12-3
12-1
12 -5

t Statistical tests were performed on the matched
pairs.

t If there was more than 1 like-sexed sib in a
family (4 instances for males, 2 for females) mean
values for sibs in same family were used in calculat-
ing mean for the group of families.

differences reaches a level of significance,
but it is noteworthy that for each para-
meter the value for male patients is lower
than that of their brothers. The numbers of
individuals involved, however, is small.
AML patients

In the dermatoglyphic analysis sum-
marised in Tables I, II and III, the 19
boys with AML do not differ significantly
from the controls, or from the patients
with ALL. The group of 12 girls, however,
have significantly greater TRC, PIF,
numbers of digital whorls, hypothenar
pattern and hypothenar loop H than girl
controls.

Grouping the patients according to
cytological type, age or survival did not
reveal any differences in the distribution of
dermatoglyphic features, but it should be
noted that the number of patients is small.

AML families

Parents.-The dermatoglyphic findings
in AML parents are also shown in Tables I,
II and III. Those of the AML fathers differ
little from controls. The mean values for
TRC, digital whorls, and hallucal tibial
loops are higher in AML mothers than in
control mothers, but only in tibial loops
does the difference reach statistical signi-
ficance.

Only 6 of the male and one of the female
AML patients had like-sex sibs, making
comparisons of little value.

Significantly (P<0.05) more first-degree
relatives of AML patients (66%) than
control subjects (50%) bore hypothenar
patterns on at least one hand. This was
mainly due to the increased prevalence in
AML families of the common loop H
(Lr). A rare form of this loop H!, designa-
ted LrA by Weninger (1947) and described
by her as occurring in 0.76% of normal
males and 1.25% of normal females, was
later reported as occurring in 6.4% of male
schizophrenics (Pons, 1959). This pattern
was not found in our series amongst 295
members of the control families, nor
among 234 members of families of ALL
patients, but was present in one ALL
patient and in 5 individuals (2 male
patients, one father, one brother and one
sister) in 5 different families of AML
patients (Table IX). One of the affected
AML patients survived 127 weeks after diag-
nosis (the median survival for the group
being 69 weeks) and the other is well and
off treatment more than 4 years since diag-
nosis. The ALL patient is still in her first
remission, 5 years since diagnosis.

DISCUSSION

ALL

Analysis of the dermatoglyphic prints
of this series of 152 children with ALL and
181 first-degree relatives of 54 of them
shows few differences from control individ-
uals, and these not of convincing signifi-
cance, and thus does not support the
suggestion that, except in the case of
Down's Syndrome, a congenital suscept-

1071

M. TILL, S. LARRAURI AND P. G. SMITH

TABLE IX.-Prevalence of Hypothenar Loop H! in PatIents, their First-degree

Relatives and Controls

No.

AML

Families

Family members (including

patients)
Patients*
ALL

Families

Family members (including

patients)
Patients*
CONTROLS

Families

Family members

25

103

31

54
234
152

75
295

Fisher's Exact

Hypothenar     Test (vs. controls)

loop H!        (2-sided prob)

5
5
2

0001
0 * 002
0 *02

0

0
1

0 7

0
0

* Includes patients whose families were not studied. Statistical comparion is made with control family
members.

ibility to ALL might be identified by
means of dermatoglyphics. The absence of
the normal sex difference for PIF and TRC
and the reversed sex incidence of digital
arches in ALL patients, although not
significant, supports in some measure the
findings of Purvis-Smith and Menser
(1973a) but the lack of increased incidence
of digital whorls in patients is in disagree-
ment with these authors.

The subjective element involved in
reading abnormalities of the palmar creases
and the various ways of reporting them
makes direct comparison between pub-
lished reports difficult. In the series
reported here, where the prints were
examined more than once without know-
ledge of the identity of the subjects, the
prevalence of simian lines agrees reason-
ably well with the findings of Purvis-
Smith and Menser (1973a) and Wertelecki
et al. (1973) but the frequency of Sydney
lines is greater, particularly in control
groups, than that found by those authors.
There is agreement with both the previous
reports that anomalous creases occur as
often in the patients' sibs as in the
patients and also with Purvis-Smith and
Menser (1973a) that anomalous creases of
any type occur more often in the children
of parents, who have anomalous creases
than in those whose parents have normal
creases; but since these findings are equally

applicable to control families (Table VII)
they cannot be associated with a genetic
predisposition to ALL.

The possibility that some environmental
factor affecting early foetal development,
and in particular the development of the
skin ridges, might be of aetiological
importance in ALL arose following reports
that subjects with congenital rubella have
unusually high mean values for digital
whorls and PIF (Alter and Schulenberg,
1966; Purvis-Smith and Menser, 1973b).
The finding that male patients with ALL
had more finger-tip pattern than their like-
sex sibs (Wertelecki et al., 1973; Purvis-
Smith and Menser, 1973a) supported this
possibility, but a numerically similar
group of male ALL patients with brothers
in the series described here, show quite
opposite results. Since none of the differ-
ences reported here between ALL patients
and their brothers is statistically signifi-
cant, and there are no marked differences
between female patients and their sisters,
there is no support for the hypothesis that
an antenatal event which is of aetiological
importance in ALL also affects the
development of dermatoglyphic features.

Since development of the thymus and
the immune system and that of the skin
ridges both commence during the third
month of foetal life, it is tempting to argue
that factors affecting the development of

1072

DERMATOGLYPHICS IN CHILDHOOD LEUJKAEMIA        1073

one of these sytems may affect both.
Since the ALL patients described here all
received very similar treatment, the fact
that their dermatoglyphic features were
associated with response to treatment
might be attributable to an underlying
association of dermatoglyphics with the
efficiency of the immune response.

The variation in results reported by
different authors may be partly explained
(as emphasised by Berka et al., 1971) by
the large number of dermatoglyphic
features available for comparison. No
constant distinguishing characteristic for
ALL patients is found in every series and
there is no strong suggestion of a genetic
susceptibility for ALL. The results re-
ported here relating the association of
dermatoglyphic features with prognosis
need to be tested in other series but, if
confirmed, might have clinical application
in choosing treatment protocols. The
extent to which these results could explain
the discrepancies between the various
reports cannot be assessed, since most
authors do not detail how the patients were
sampled.
AML

In contrast to the results in ALL, the
greatly increased incidence of the rare
loop H! in AML patients and their first-
degree relatives suggests the presence of a
genetically determined factor carrying a
high risk of AML. There is also a suggestion
that this pattern might be associated with
good prognosis in individuals developing
leukaemia.

The significantly raised PIF and digital
whorls in girls with AML are as high as
those quoted for girls with congenital
rubella (Alter and Schulenberg, 1966;
Purvis-Smith and Menser, 1973b) but
interpretation of this finding is difficult,
since the number of patients is small and
only one had a sister for comparison. The
finding that the boys with AML show no
such distinguishing features, however, is

against the suggestion of an antenatal
environmental cause, as is the finding that
mothers of AML patients resemble the
female patients to a limited extent in
having a higher incidence of both digital
whorls and hypothenar pattern than
control mothers.

Dermatoglyphics have previously been
reported only in very small numbers of
children with AML and the series reported
here is only large enough to allow tentative
conclusions. However, the increased fre-
quency of the loop H! in these patients and
their families is striking and suggests that a
study of other genetic markers, such as
HLA, might be of interest in such families.

Dr Judith Chessells kindly provided us with ready
access to her patients and their families. We are
grateful to Professor P. E. Polani for his advice and
suggestions and to Professor R. M. Hardisty for
continued support and encouragement. We would
like to thank Barbara Crossley who gave consider-
able help with the computing and Jean Bridger for
typing the manuscript.

The work was supported by grants from the
Leukaemia Research Fund.

REFERENCES

ALTER, M. & SCHIJLENBERG, R. (1966) Dermatogly-

phics in the Rubella Syndrome. J. Am. med. Ass.,
197, 93.

BERKA, L., MCCLURE, P. D., SONLEY, M. J. &

THOMPSON, M. W. (1971) Dermatoglyphics in
Childhood Leukaemia. Can. med. Ass. J. 105, 476.
CUMMINs, H. & MIDLO, C. (1943) Finger Prints,

Palms and Soles. An Introduction to Dermatogly-
phics. New York: Dover Publications.

PENROSE, L. S. & LOESCH, D. (1969) Dermatogly-

phic Sole Patterns, a New Attempt at Classifica-
tion. Hum. Biol., 41, 427.

PENROSE, L. S. & LOESCH, D. (1970) Topical

Classification of Palmar Dermatoglyphics. J.
ment. Def. Res., 14, 111.

PoNs, J. (1959) Relaciones entre Esquizoprenia y

Lineas Dermapapilares. Genetica Iberica, 11, 1.

PIJRVIS-SMITH, S. G. & MENSER, M. (1973a) Derma-

toglyphics in Children with Acute Leukaemia. Br.
med. J. ii, 646.

PURVIS-SMITH, S. G. & MENSER, M. (1973b) Genetic

and Environmental Influences on Digital Derma-
toglyphics in Congenital Rubella. Pediat. Res., 7,
215.

WENINGER, M. VON (1947) Zur Vererbung der

Hautleistenmuster am Hypothenar der Mensch-
lichen Hand. Mitt. Ost. Ges. Anthrop., 73, 55.

WERTELECKI, W., PLATO, C. C., FRAUMENI, J. F. &

NISWANDER, J. D. (1973) Dermatoglyphics in
Leukemia. Pediat. Res., 7, 620.

				


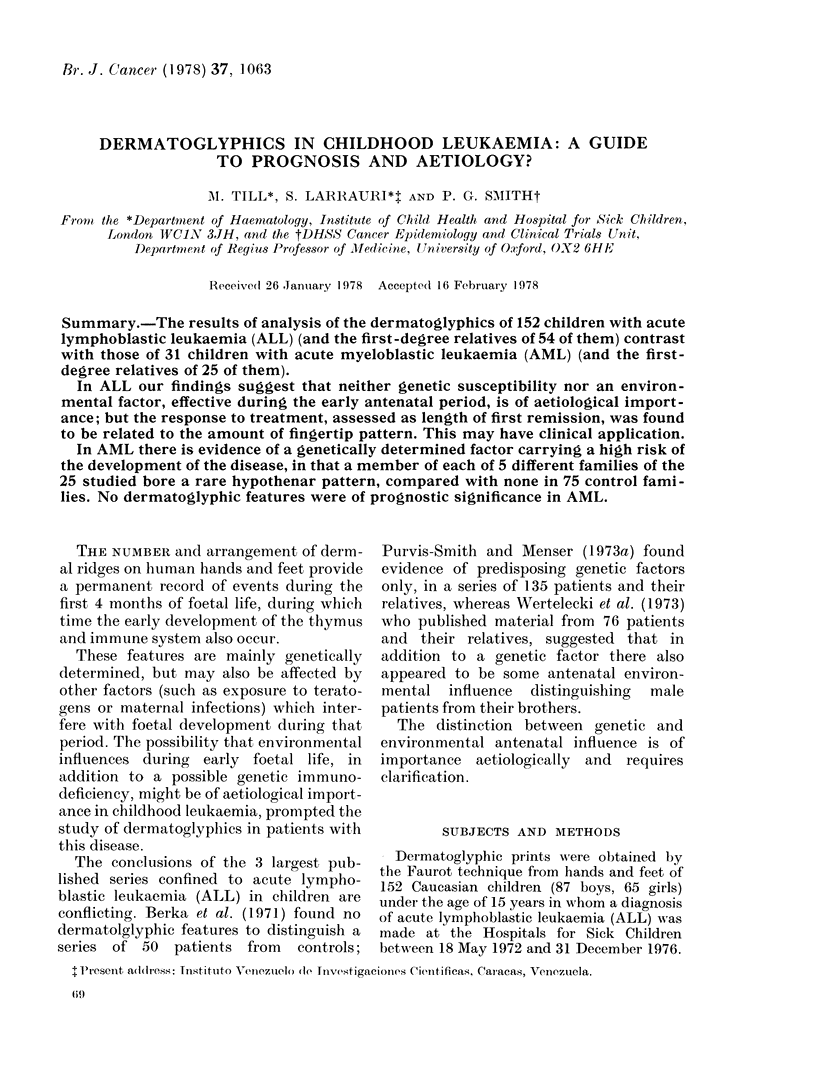

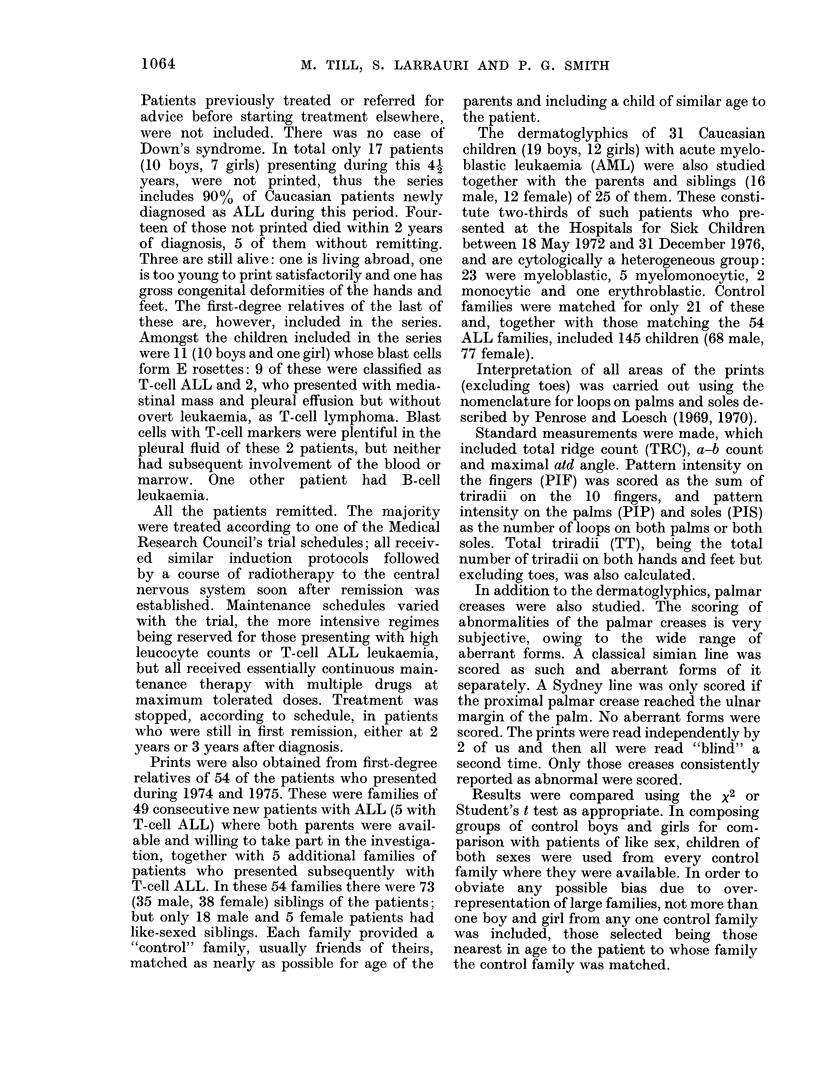

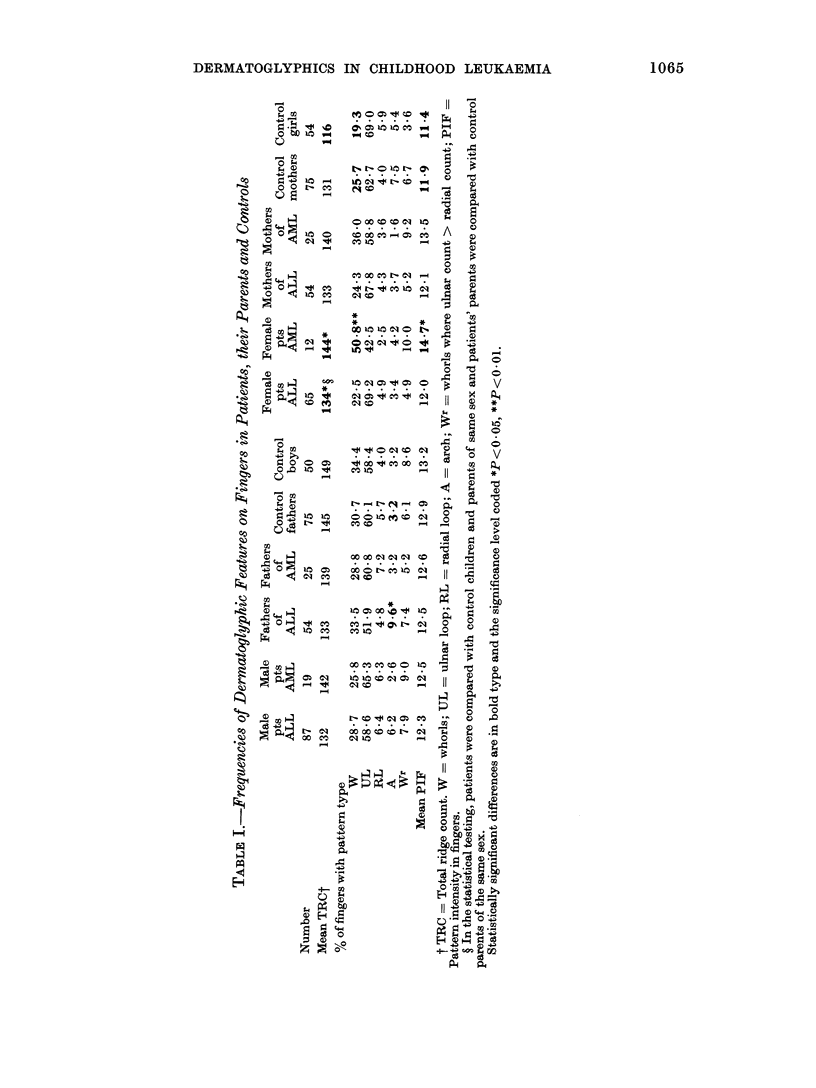

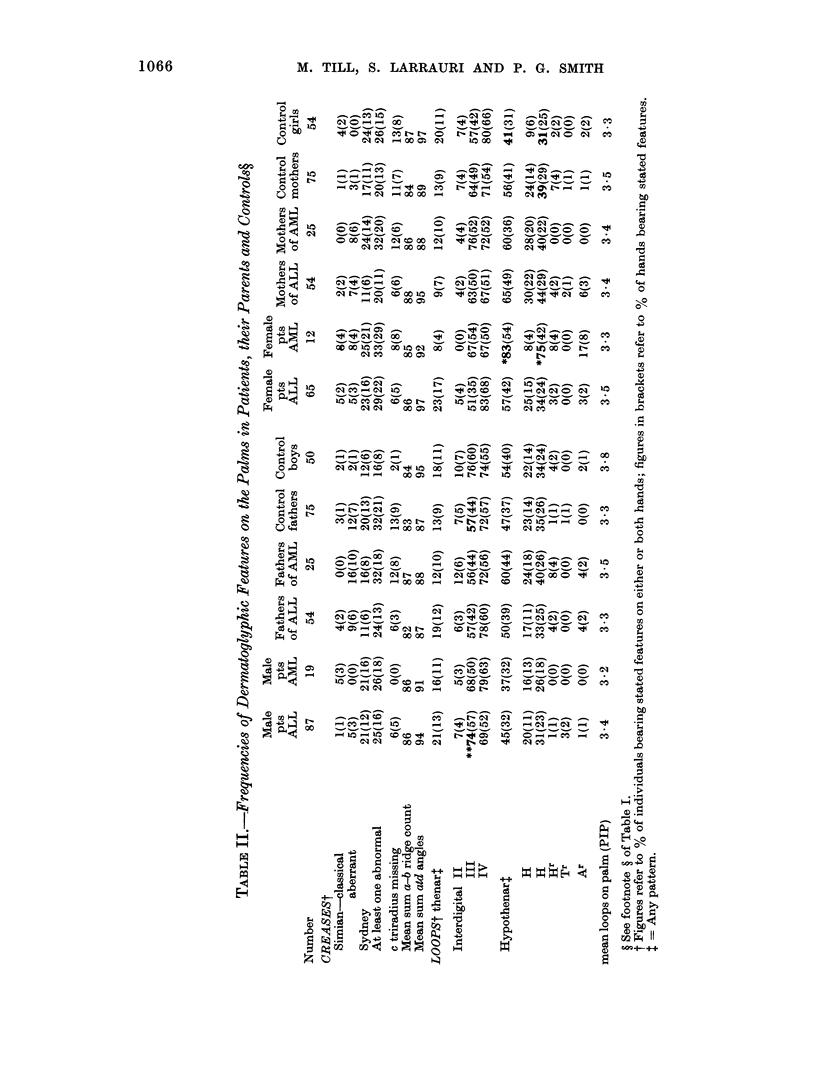

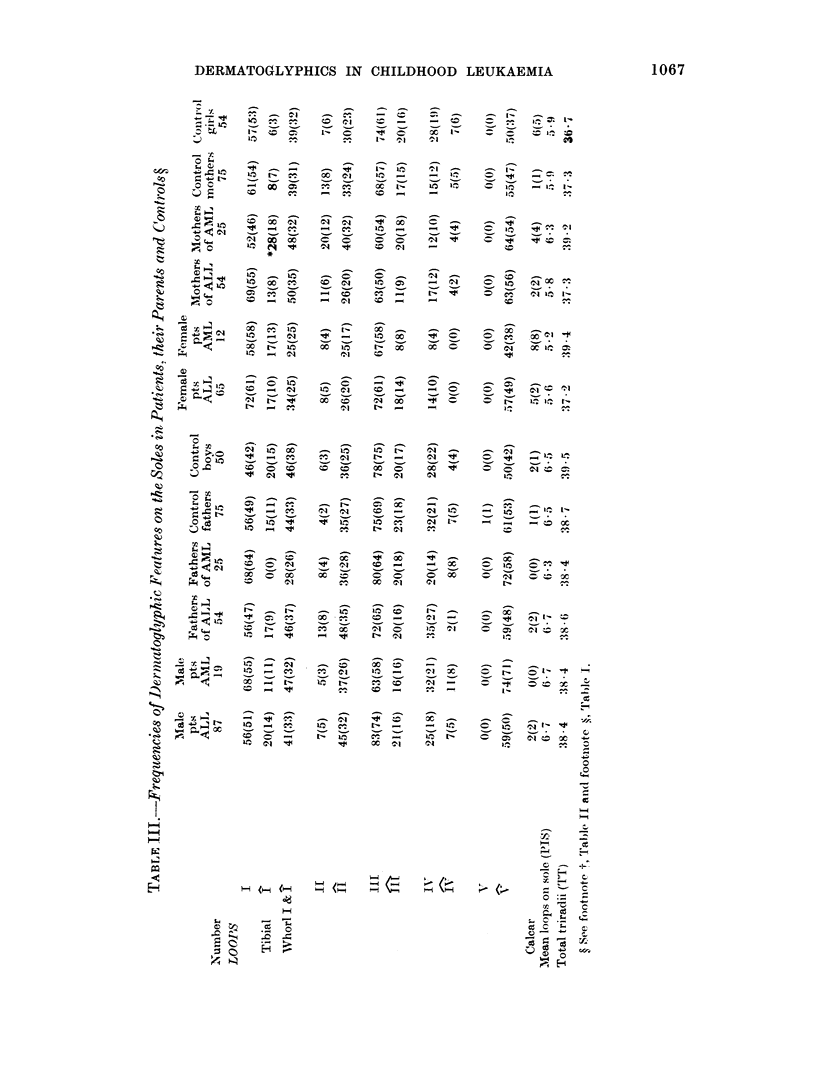

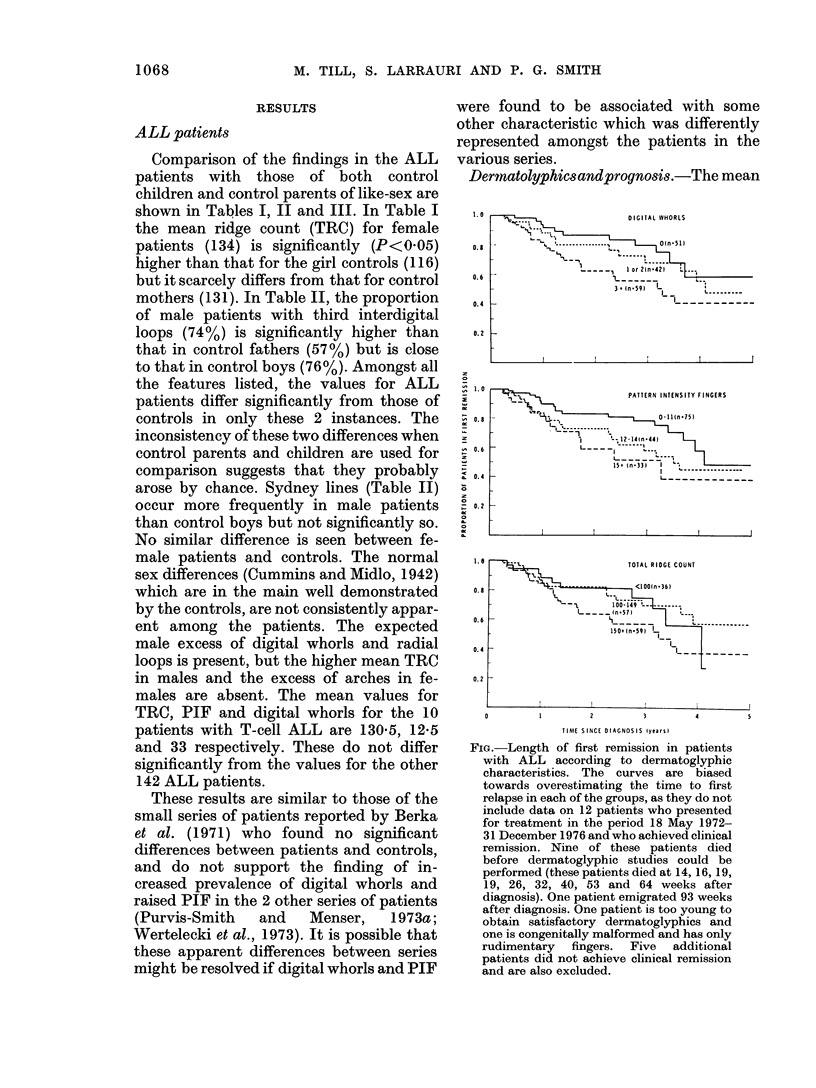

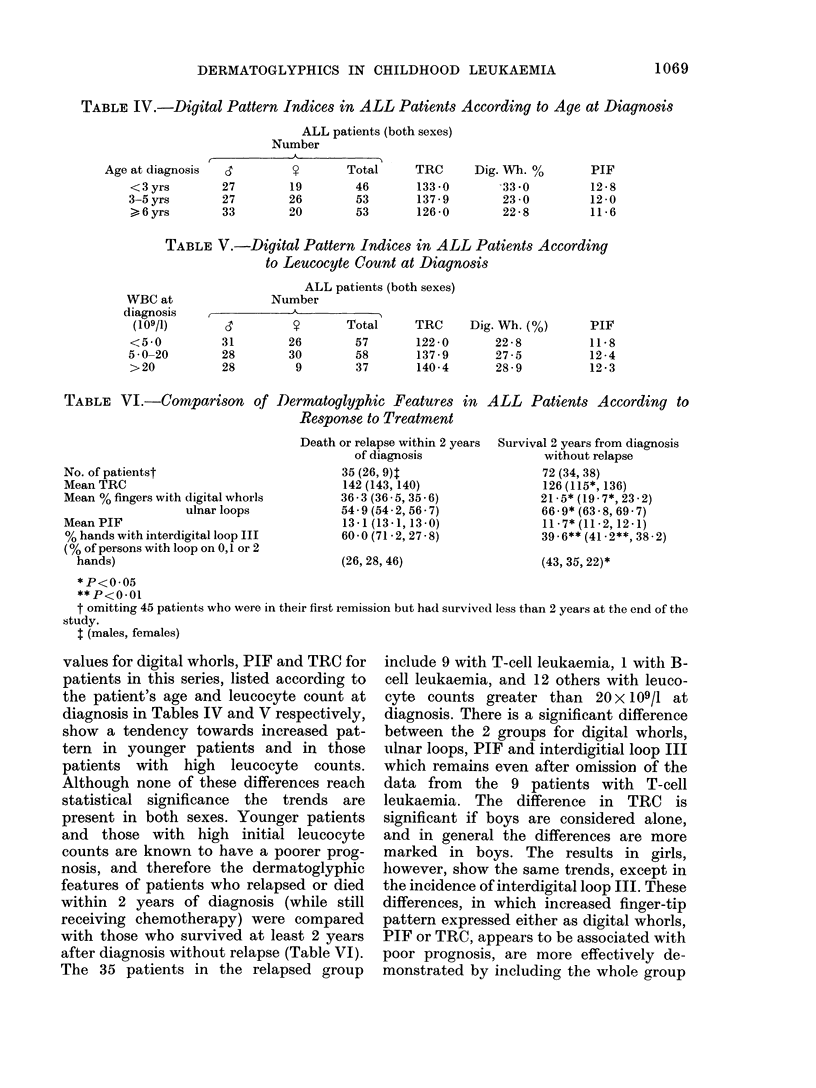

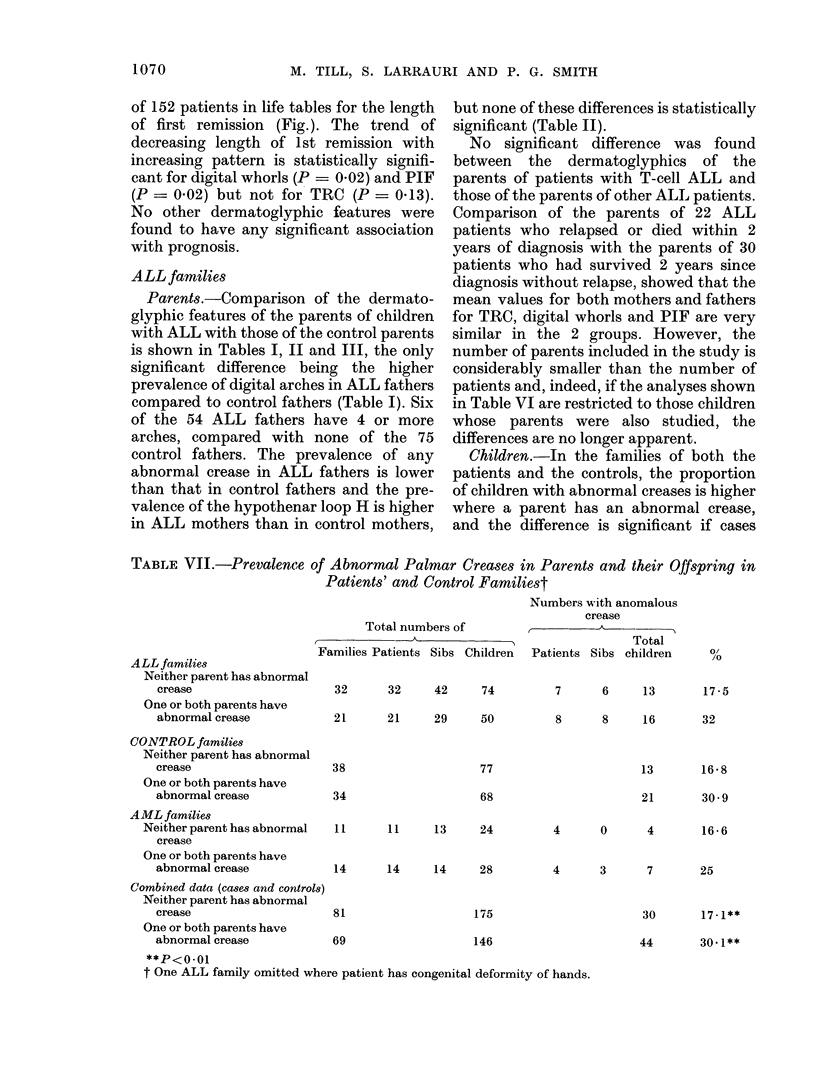

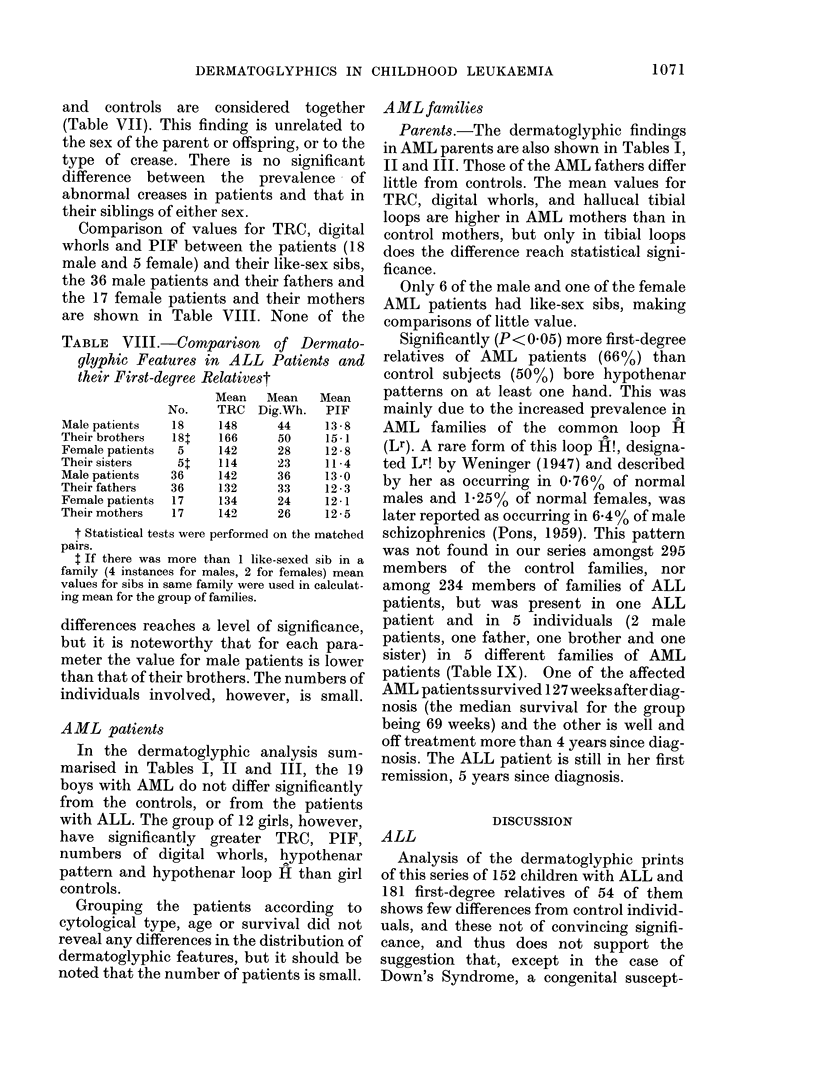

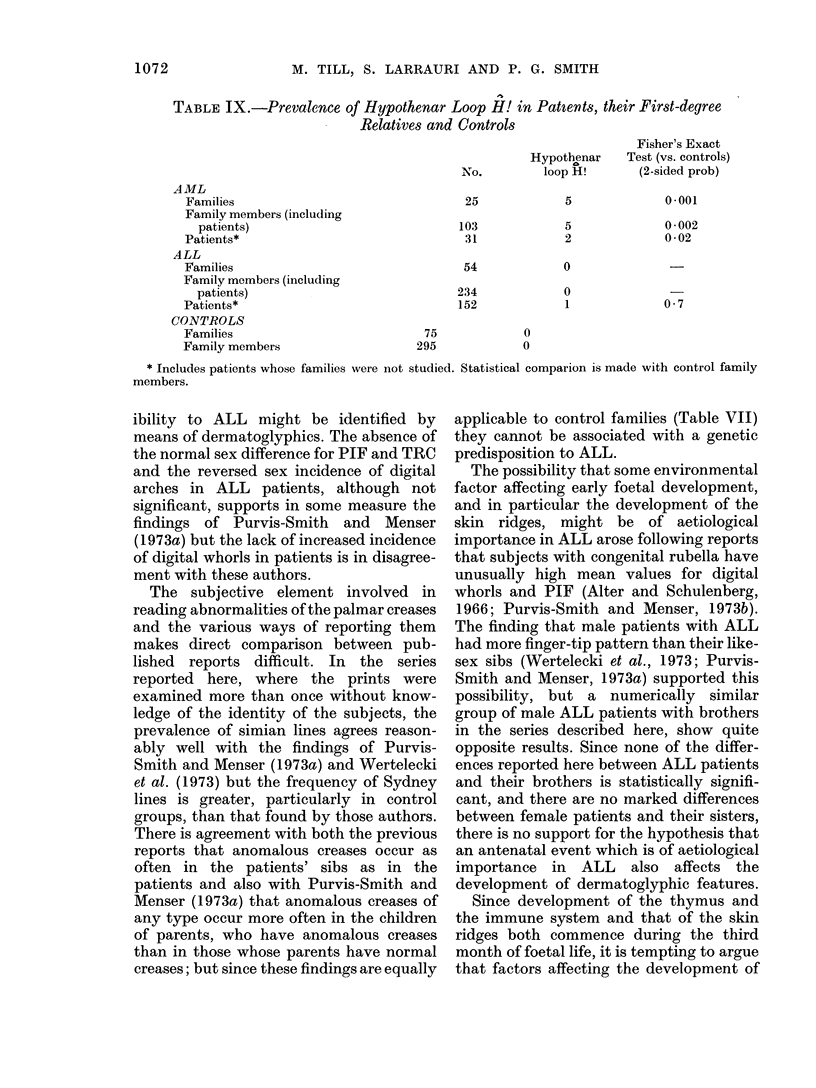

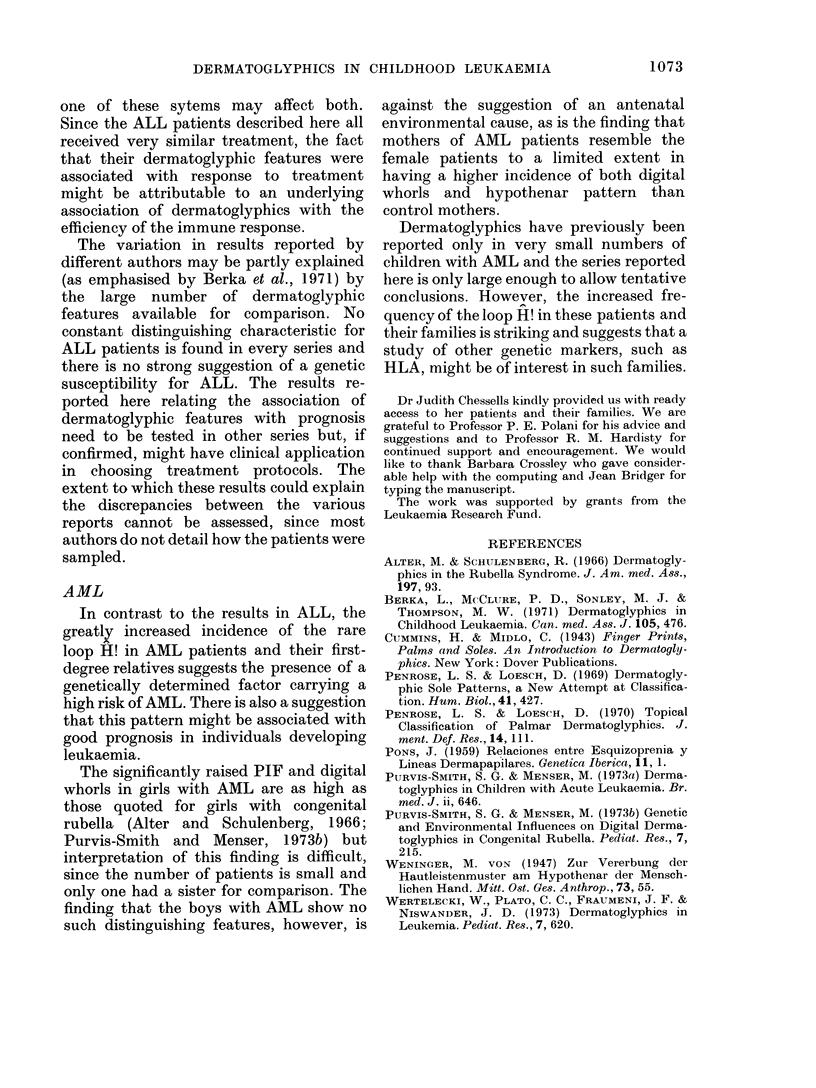

